# Making the case for a fracture liaison service: a qualitative study of the experiences of clinicians and service managers

**DOI:** 10.1186/s12891-015-0722-z

**Published:** 2015-10-01

**Authors:** Sarah Drew, Rachael Gooberman-Hill, Andrew Farmer, Laura Graham, M Kassim Javaid, Cyrus Cooper, Andrew Judge, The REFReSH study group

**Affiliations:** Oxford NIHR Musculoskeletal Biomedical Research Unit, Nuffield Department of Orthopaedics, Rheumatology and Musculoskeletal Sciences, University of Oxford, Windmill Road, Headington, Oxford, OX3 7LD UK; School of Clinical Sciences, University of Bristol, Learning and Research Building, Level 1, Southmead Hospital, Bristol, BS10 5NB UK; Nuffield Department of Primary Care Health Sciences, Radcliffe Observatory Quarter, Oxford, OX2 6GG UK; Neurosciences, Trauma and Specialist Surgery, Oxford University Hospital Trust, John Radcliffe Hospital, Oxford, OX3 9DU UK; MRC Lifecourse Epidemiology Unit, University of Southampton,, Southampton General Hospital, Southampton, SO16 6YD UK

**Keywords:** Osteoporosis, Hip fracture, Commissioning, Business cases, Qualitative research

## Abstract

**Background:**

To develop services, healthcare professionals must make business cases to managerial bodies within Hospital Trusts and if approved, to commissioning bodies. Patients with hip fracture are at high risk of subsequent fracture. To prevent this, guidance recommends structuring fracture prevention services around coordinator based models. These are known as Fracture Liaison Services (FLS).

**Methods:**

33 semi-structured qualitative interviews were conducted with healthcare professionals with experience of making business cases for FLS. Data was analysed thematically.

**Results:**

Challenges in the development of business cases included collecting all the relevant data and negotiating compartmentalised budgets that impeded service development. Participants described communication and cooperation between providers and commissioners as variable. They felt financial considerations were the most important factor in funding decisions, while improved quality of care was less influential. Other factors included national guidelines and political priorities. The personalities of clinicians championing services, and the clinical interests of commissioners were seen to influence the decision-making process, suggesting that participants felt that decisions were not always made on the basis of evidence-based care. Effective strategies included ways of providing support, demonstrating potential cost effectiveness and improved quality of care. Using a range of sources including audit data collected on the successful Glasgow FLS, and improving cooperation between stakeholders was advocated. Participants felt that the work of commissioners and providers should be better integrated and suggested strategies for doing this.

**Conclusions:**

This study provides information to healthcare professionals about how best to develop business cases for FLS. We conclude with recommendations on how to develop effective cases. These include using guidance such as toolkits, aligning the aims of FLS with national priorities and benchmarking services against comparators. Introducing a ‘Local Champion’ to work alongside the service manager and establishing a multi-disciplinary working team would facilitate communication between stakeholders. Involving commissioners in service design would help integrate the roles of purchasers and providers.

## Background

Commissioning (purchasing) processes in the UK’s National Health Service (NHS) are founded on a division between purchasers and providers [1]. In April 2013, responsibility for the commissioning of services changed from the Department of Health and local Primary Care Trusts led by managers, to NHS England, an organisation overseeing 211 GP led local Clinical Commissioning Groups [2]. Foundation Trusts are independent organisations responsible for providing over half of NHS secondary care services operating in local regions. They are contracted to deliver services commissioned by local Clinical Commissioning Groups and NHS England, and all NHS Trusts are in the process of becoming Foundation Trusts [3]. Commissioning processes have been targeted by the Department of Health as a means of safe-guarding standards of care and driving quality improvements in the NHS [4]. Core principles outlined by the NHS Commissioning Board states funding decisions should be based on the cost-effectiveness of the proposed service, clinical outcomes and the strategic plans of the NHS Trust. Decisions should therefore be made on the strength of the available evidence and the robustness of the business cases [2].

Within the new commissioning structures all proposals for new services must be made through submission of business cases [2]. These are considered by managerial bodies within the local NHS Trust, which include senior clinicians and managers. If supported, then the case for a new service is presented to their Clinical Commissioning Groups for approval. Responsibility for developing business cases rests with clinicians and operational service managers. Finance managers develop budgets for new services and patient representatives, clinicians and service managers from other departments may also be involved in case development.

Previous research exploring the day-to-day experiences of commissioning in the UK has been largely focused on commissioners, highlighting experiences of those working under the Primary Care Trusts [5–7] and to a lesser extent, the newly formed Clinical Commissioning Groups [8, 9]. These have identified factors that inform commissioning decisions, including cost-effectiveness [7, 10], clinical guidelines and benchmarking data [7] . They also suggest that the relationships between stakeholders involved in the commissioning process are variable [6, 11, 12]. More recent research has also examined the attitudes of GP providers towards the introduction of Clinical Commissioning Groups, indicating that GPs are concerned that they lack the capacity and capability to engage in commissioning [13, 14]. However, with the exception of Shaw et al. [15], who suggest that there are high levels of integration between commissioners and providers in commissioning processes, research exploring the experiences of acute providers has been limited.

Hip fracture is an example of an important health problem that necessitates specialised services. Each year 87,000 hip fractures occur in the UK [16] at a cost of around £2.3 billion [17]. Hip fractures usually occur when individuals with underlying osteoporosis fall [17], and patients who have a hip fracture are at high risk of further fragility fractures and premature death [18]. Effective management of these fractures can reduce the risk of further fractures [16]. Guidance recommends structuring fracture prevention services around a dedicated coordinator who provides a link between all the multi-disciplinary teams involved, an approach known as a Fracture Liaison Service (FLS) [16]. The model proposed by the Department of Health is one coordinated by a nurse specialist with support from a lead clinician in osteoporosis [19]. The efficacy of this model has been supported by a number of studies [20]. Despite this, fewer than 40 % of hospitals have established a FLS in the UK [21]. In the new commissioning structures, provision of any new services necessitates development and approval of business cases. There is already some support in place for clinicians and service managers who wish to develop business cases for a FLS, including advice about how to establish a dedicated multi-disciplinary team to lead the case and business case templates [22, 23]. Although practical, the advice is not based on an understanding of issues that clinicians and service managers themselves see as relevant to the production of strong business cases with good chance of success. Identifying these has the potential to inform the development of further guidance and recommendations for development of business case specifically for FLS. More generally, understanding the views of clinicians and service managers involved in the delivery of services of making business cases provides us with valuable information about how new commissioning processes are experienced by these groups.

The study aims to: a) explore the experiences of clinicians and service managers of developing and making business cases for a FLS; b) identify factors that are seen to inform the decision of managerial bodies to approve a FLS; c) describe clinicians’ and service managers’ views about the best ways of making business cases for this service.

## Methods

### Sample

This qualitative study took place in 11 acute hospitals that receive hip fracture patients in a region of England, as part of a larger study exploring hip fracture services [24]. Clinicians and service managers involved in planning and delivering fracture prevention services were approached about participation. Potential participants were identified by a Clinical Lead Champion in Osteoporosis and purposively sampled to ensure inclusion of professionals from all 11 hospitals.

In three waves of recruitment, potential participants were approached first by email then followed-up by further email and telephone calls. Snowball sampling was used to widen sampling [25], with participants suggesting other individuals to contact. In total, 82 healthcare professionals were contacted and 43 agreed to take part in qualitative interviews. The remainder either declined or were unavailable. Of these, 33 participants had experience of making business cases for a FLS and it is these who are included in our analysis. The sample size reflects inclusion of a range of professionals from all hospitals, their availability and knowledge of business case development. Rather than aiming to achieve data saturation [25] the study sought to generate sufficient and adequate material reflecting appropriate range of experiences [26].

### Ethical approval

Ethical approval was provided by the Central University Research Ethics Committee (CUREC) in 2012, reference number MSD-IDREC-C1-2012-147. Written, informed consent was provided by all participants prior to interview. Each involved NHS Trust provided R&D approval for the study.

### Interview procedure

The qualitative researcher (SD) undertook face-to-face interviews in 2013 lasting between 30 and 50 min. A topic guide helped the researcher to explore participants’ experiences of making business cases for a FLS, factors that they felt informed the decision of commissioners to approve the new service and what they considered to be the best strategies for making them. The interviewer used methods such as ‘probing’ to help achieve depth in the data [27]. Interviews were piloted with three participants in order to refine the topic guide and the three pilot interviews are included in the dataset.

### Data analysis

Interviews were audio-recorded, transcribed, anonymised and imported into the data analysis software NVivo. Using a thematic approach to analysis [28], transcripts were coded and then these grouped to identify dominant themes and subthemes. Emerging themes were compared and contrasted with the data extracts and transcripts to ensure interpretations reflected the data collected, with memos to record thoughts and ideas as themes developed. 20 % of the interview transcripts were independently coded by another member of the team (RG-H) and codes discussed to arrive at a single code list [29]. To enable cross-comparison, coded data were displayed on charts using the framework approach to data organisation [30]. Identifying features have been removed from the data presented in Tables 1, 2 and 3.

**Table 1:** Experiences of making business cases for an FLS

*[Making business cases is a] total and utter brick wall… and after a while you do lose the will to live.* [Participant ID: 029]
*I will never be able to write a good business case, because I can’t. I don’t like doing them, I get bored, it’s not where my skills are so don’t ask me to do it.* [Participant ID: 009]
*You constantly find you’ve got to go digging around for statistics. You’re trying to find numbers from data that isn’t collected… [it’s] all done on percentages and sort of guesswork.* [Participant ID: 008]
*If you start getting into areas of complexity, so frailty complexity, where you get different services crossing: whose budget is it? It’s harder to cost them… just things like this don’t fit into categories.* [Participant ID: 007]
*[The service manager's] priorities are dependent on their interests.* [Participant ID: 030] *We felt that we were setting up and driving towards an improved Hip Fracture Service… but we were doing it without the support of our management.* [Participant ID: 038]

**Table 2:** Factors seen to determine whether an FLS is approved

*The powers that be don’t want to put the money into it because you’re not going to see an instant result.* [Participant ID: 015]
*It has kind of lost the quality of care a little bit… quality doesn’t pay the bills unfortunately* [Participant ID: 023]
*What tends to happen in healthcare is that you get certain things that become a certain flavour of the month. So lets look at this, this and this disease. Right what are we going to do in hospitals, whose doing that, right we think this is a priority would you like to do something in this area.* [Participant ID: 026]
*I think it often comes down to the individual people who are doing it… you’ve got [a local champion in another hospital] sort of waving his little flag and everybody listens. Well if you hadn’t have had a him you may not have had that service.* [Participant ID: 023]
*He who shouts loudest gets most* [Participant ID: 001]
*We have this problem with commissioning the local General Practitioners, if you have a clinical lead who has a strong interest in dementia services then there will be lots of money put into dementia services.* [Participant ID: 001]
*If the chairman of a CCG had had a mother with a hip fracture (laughs) that CCG, I can tell you, someone would push it through.* [Participant ID: 008]

**Table 3:** Best ways of making a business case for a FLS

*You can use other [services as] comparators and the USA seems to be quite a good thing to beat doctors with at the moment so if you look at outcome in the best centres compared to the average centres, which is quite a good… we do this but [another Trust] do this, what’s the difference? Well they’ve got 15 Fracture Liaison Nurses and everyone doing this… [the Trust] tend to benchmark things across local groups but if you’ve got national comparators those are quite good.* [Participant ID: 021]
*I think patients’ voice can have more power, especially if we are looking at situations where we feel that patients may be at risk and I think that obviously helps to drive the changes as well.* [Participant ID: 012]
*You also need to get allies on side who will help you and support you… people who are key, for example, [a GP], she wasn’t involved in commissioning or anything but she is very well respected in her field* [Participant ID: 004]
*I think the managers are too separate and it should be more embedded and integrated so that we work together and we come up with clinical designs and models, they tell us we’re stupid anspending too much money, and then we work it out and come up with a compromise for it.* [Participant ID: 009]
*It’s through no fault of anyone’s but these are human organisations and you’ve got to engage with these things on a human level… It’s much easier to say no to someone who you’ve been saying no repeatedly to for the last five years and where you’re not regularly seeing them.* [Participant ID: 024]

## Results

### Characteristics of sample

The 33 participants comprised five fracture prevention nurses, four orthogeriatricians, four geriatricians, two GP osteoporosis specialists, three consultant trauma orthopaedic surgeons, six rheumatology consultants, two orthopaedic nurses, one trauma matron, one bone densitrometry specialist and five service managers. Between one and five participants were from each of the 11 hospitals. Clinicians had spent between 1 ½ and 27 years working at the hospital and had between 8 months and 43 years since qualification in their current role. Service managers, by contrast, had spent less time working at the hospital, a mean of 2 ¼ years, and had between 2 and 6 years of experience. Of the 11 hospitals included in this study, seven had established a FLS coordinated by a nurse specialist with support from a clinical lead by 2013.

### Experiences of making business cases for a FLS (Table 1)

Clinicians and service managers found the experience challenging and a number of clinicians expressed frustration. Clinicians and managers found the process of constructing business cases time consuming and struggled to find the capacity to do this whilst fulfilling clinical commitments.

Approval processes were also described as ‘tedious’ and ‘incredibly cumbersome’. This was thought to be exacerbated by rapid turnover of operational service managers and senior managers within the Trusts that meant progress was lost, in addition to multiple tiers of management with responsibility for approval.

Some clinicians felt more capable than others of making business cases and understanding the approval processes, based on their individual skillsets and levels of training. A number of participants found it difficult to collect all the relevant data required since quality was often lacking. Others were unsure how to effectively demonstrate improvements in care quality.

Compartmentalisation of budgets within individual hospital departments was seen as a barrier to service development. Participants described lack of agreement about which Department should be responsible for funding a service, which was linked to the involvement of multi-disciplinary teams in the prevention of secondary fractures, both within the hospital and community care [16].

Participants recognised the value of effective communication and cooperation between a range of stakeholders involved in the commissioning process, but described this as variable. Within the multi-disciplinary team, participants identified examples of effective communication but had also encountered colleagues that were more ‘resistant to change’. One participant attributed this to the cynicism surrounding the process. Although participants described examples of successful co-working between clinicians and operational service managers, some service managers wanted more help from clinicians. A number of clinicians also found it difficult that service managers whom they worked with did not seem to be interested in service development. In general there was a view that communication with commissioners and providers (including Trust Managers) was variable and could be improved.

### Views about FLS approval (Table 2)

Participants discussed their views about factors considered in commissioning processes. They thought financial considerations were most important and that there was pressure to generate short-term cost savings. There was concern that this presented a challenge for fracture prevention services, which tend to generate longer-term savings.

Clinicians tended to think quality of care was afforded low priority in decision-making processes, while service managers thought that quality of care was a consideration. Participants thought that national guidelines and political priorities were key factors in decisions about services, and some expressed dissatisfaction with the importance afforded to these. Participants felt commissioners were less likely to approve FLS than other services, since osteoporosis was viewed as a low national priority and because there was a belief that osteoporosis should be managed in primary care, reflecting a more general shift of resources into primary care [31].

Clinicians thought that funding tended to be channelled into high profile ‘failing’ services that had poor clinical outcomes and audit results. Participants from one hospital were frustrated that their service had to deteriorate before they received funding for a FLS.

The ambition and personality of ‘local champions’, the clinician involved in leading the business case [32], was also seen to impact on whether the service was approved. Participants also felt the clinical interests of GP commissioners influenced the decision-making process since they were more likely to approve services that reflected their priorities. As a result, there was a suggestion that funding decisions were not always based on principles of evidence based healthcare.

### Best ways of making a business case for an FLS (Table 3)

Participants described ways they felt the challenges of business case development could be addressed. They identified the potential value of training courses, templates or ‘toolkits’. Other suggestions for enhancing support included the introduction of mentorship schemes with those who had successfully set up services, or establishing a network of mutually supportive service managers and clinicians.

Participants suggested using empirical evidence in business cases to demonstrate the efficacy of different models of care. For instance, some advocated citing research on the Glasgow FLS, a service that has contributed to a 7.3 % reduction in hip fractures in the city over 10 years, compared to rise of 17 % across England [19]. In light of participants’ view that osteoporosis was often afforded low priority, two suggested that the status of the service could be improved if the business case emphasised how the aims of the FLS supported national health priorities identified by the Department of Health such as dementia [33].

Participants also highlighted the use of practical sources of evidence such as outcomes data, like the Hospital Episodes Statistics [34], to make cost effectiveness calculations. Benchmarking the service using clinical outcomes data against local and national comparators was advocated. Some participants suggested involving the public, either through patient satisfaction questionnaires or patient representatives, although few had done so in practice.

To develop effective communication and cooperation, participants advocated contacting all those involved in developing the case early on to garner support and ensure the case was viable. Service managers felt that clinicians had an important role in canvassing the support of their colleagues and one felt that regular meetings with the Clinical Lead had already helped them to work together successfully. Building networks with the ‘right’ people, such as prominent clinicians who could support the case, was described as a helpful way to achieve respect from commissioners.

Participants thought that the roles of purchasers and providers should be more closely integrated. Strategies included approaching managerial bodies early in the process to help establish their priorities and give them ‘advanced warning’ of funding requests. Developing plans with Clinical Commissioning Groups and informal networking were also suggested.

## Discussion

This study has identified and explored clinicians’ and managers’ experiences of making business cases for FLS, factors that are seen to determine whether the case is approved and views on the most effective ways of making these business cases. Participants found the process of business case development to be time consuming and frustrating, with collection of relevant data a challenge. Participants thought that competence and understanding of processes varied. The current funding allocation mechanisms within the NHS were seen to make funding a service across several disciplines harder to achieve. Communication and cooperation between the stakeholders involved in these processes, including providers and commissioners, was seen as important but not always optimal. Participants described their views about factors that influenced funding approval. They thought financial considerations were afforded most importance, and other factors included national guidelines and political priorities. Clinicians thought improvements to care quality were not a priority, but this view was not echoed by managers. The personality and drive of local clinical champions, and clinical interests of commissioners were also seen to influence funding decisions and that as a result, decisions were not always based on the principles of evidence based healthcare. Finally, participants identified ways in which business case development could be supported. Suggestions included provision of training courses and the introduction of mentorship schemes. The importance of using empirical evidence such as clinical guidelines and academic research as well as practical sources of evidence was highlighted. Strategies for improving communication and cooperation between stakeholders were also suggested.

Previous studies exploring providers’ experiences of commissioning under the Primary Care Trusts found the processes time consuming [15] and collecting all relevant data difficult since available data was often of low quality [15, 35]. The challenge of managing the relationship between purchasers and providers has also previously been highlighted [11, 12, 36]. Although one study found that there were high levels of cooperation [15], others, as with ours, concluded that the relational aspects of commissioning could be improved [11, 12]. Our study also succeeds in communicating the lack of confidence of providers in navigating these processes and the difficulty of funding services across several disciplines.

Factors that participants identified as informing commissioning decisions reflect guidance issued by the NHS Commissioning Board [37]. However, participants felt that commissioners placed different relative emphasis on these factors. Participants also questioned the objectivity of the Clinical Commissioning Groups, suggesting the personality of local clinical champions and the clinical interests of commissioners impacted on decision-making. The potential influence of clinical interests supports concerns raised by participants in previous studies [35, 38]. Although the introduction of GP-led Clinical Commissioning Groups were intended to involve clinicians in making funding decisions then [2], this suggests that there may be a disconnect between the views of GP commissioners and providers.

Strategies identified to support development of effective business cases support previous work.

Using multiple sources of evidence is advocated in a business case template developed to support the implementation of a FLS. This recommends calculating the financial burden of fragility fractures for the Hospital alongside potential savings, referencing the economic burden of secondary fractures in the UK, and citing national policy and research on existing models of care [22]. The value of using multiple evidence bases is supported by findings from other studies [10, 15]. Ensuring that effective communication exists between all service providers, including those in secondary and primary care, has also been highlighted [23]. There is a strong emphasis on the need to develop multi-disciplinary working groups when developing a case, with a Clinical Lead to guide it [22, 23]. These recommendations are supported by the Fracture Liaison Implementation Programme (FLIP), a new supportive tool for designing business cases for FLS [39].

In accordance with this research, participants in previous studies have suggested that better integration between commissioners and providers may help to facilitate service development [15]. To do this, guidance advises contacting commissioners from the outset to establish a funding remit and discuss the potential scale of the service [23]. Another strategy may be to introduce representatives from commissioning bodies into these multi-disciplinary groups [22]. In addition to these factors, other recommendations include the introduction of protected time for Lead Clinicians to work on business cases [22]. This could address the lack of time many participants identified as a barrier to case development.

The use of qualitative methods enabled us to undertake a detailed exploration of the opinions and experiences of professionals. Robust analysis processes provide confidence that the data has been thoroughly explored, for instance we used double coding and team work to discuss results during the analysis process [40]. The study aimed to include participants with diverse and relevant experiences rather than to achieve data saturation; inclusion of 33 participants with experience of business case development is a strength. However, we were only able to include five service managers and although we tried to include many more we found it hard to recruit this group, which limits our ability to draw firm conclusions about their views. Despite this, the inclusion of 28 clinicians in this study is of great value because healthcare professionals are increasingly expected to develop business cases for services within the NHS. Since this work forms part of a larger interview study, it is unlikely participants self-selected on the basis of their experiences of making business cases, and we are relatively confident that the findings are not unduly influenced by selection bias. We found that there was little difference between the views of participants who had successfully established a service and those who had not. As a result, we do not distinguish between the two groups in our analysis. The study elicited verbal descriptions of the experience of making business cases, which are recollections of process. An alternative study design would have been to shadow professionals though the process, but this might have been difficult for reasons of practicality and resource. For this reason we chose interviews as a way of efficiently collecting data, and used face-to-face interviews to enable rapport between interviewer and participants. The study is also limited as only one region was included. However, the homogeneity of experiences and the inclusion of all NHS Trusts in that region indicate that it is likely that experiences described resonate with those in other areas and are transferable [25].

Further research could examine the experiences of primary care professionals in developing cases for these services, because primary care has an important role in preventing secondary fractures [16]. In addition, as FLS is a global phenomenon, it may be of use to explore experiences of commissioning services in other care settings. Doing so would provide professionals with information on how to make effectives cases in other contexts.

## Conclusions

The study provides information to healthcare professionals and service managers about how best to develop business cases for a FLS in the future. Based on these findings and in light of previous studies, we have generated a set of recommendations for developing business cases (Table 4). These findings may be of use for stakeholders involved in making business cases for other conditions.

**Table 4:** Recommendations for developing business cases for FLS

1. Using all available support. This includes national toolkits for FLS outlined by the IOF Capture the Fracture Initiative [41], the Fracture Liaison Implementation Programme (FLIP) [39], the Osteoporosis Service Development model [42] and training courses [22]. More general guidance issued by Clinical Commissioning Groups may also be used [43].
2. Using empirical evidence such as academic research to demonstrate the efficacy of FLS alongside clinical guidelines. Research identifying outcomes of the Glasgow FLS may be of use here [44]. Aligning the aims of the FLS with national priorities such as dementia could also be considered.
3. Using evidence such as outcomes data from the Hospital Episode Statistics [34], to make cost effectiveness calculations.
4. Benchmarking the service against local and national comparators using audit data.
5. Identifying a ‘Local Champion’ [22], generally the Lead Clinician within the Department, to work alongside the service manager. This clinician has an important role in providing clinical input and obtaining support from clinicians working both within and outside of the team.
6. Developing effective communication and cooperation with stakeholders working both inside and outside of the Department by establishing a multi-disciplinary working team [22]. Service managers have a responsibility to communicate with managers working in related areas.
7. Approaching managerial bodies and Commissioners early in the process to establish their priorities and working with them to develop the service if possible. Informal networking may be of use.
